# Cardiovascular outcomes after initiating GLP-1 receptor agonist or basal insulin for the routine treatment of type 2 diabetes: a region-wide retrospective study

**DOI:** 10.1186/s12933-021-01414-3

**Published:** 2021-11-13

**Authors:** Enrico Longato, Barbara Di Camillo, Giovanni Sparacino, Lara Tramontan, Angelo Avogaro, Gian Paolo Fadini

**Affiliations:** 1 Department of Information Engineering, University of Padova, 35100 Padova, Italy; 2 Arsenàl.IT, Veneto’s Research Centre for eHealth Innovation, 31100 Treviso, Italy; 3 Department of Medicine, University of Padova, Via Giustiniani 2, 35100 Padova, Italy

**Keywords:** Observational, Real world, Effectiveness, Guidelines, Pharmacotherapy

## Abstract

**Aim:**

We aimed to compare cardiovascular outcomes of patients with type 2 diabetes (T2D) who initiated GLP-1 receptor agonists (GLP-1RA) or basal insulin (BI) under routine care.

**Methods:**

We accessed the administrative claims database of the Veneto Region (Italy) to identify new users of GLP-1RA or BI in 2014–2018. Propensity score matching (PSM) was implemented to obtain two cohorts of patients with superimposable characteristics. The primary endpoint was the 3-point major adverse cardiovascular events (3P-MACE). Secondary endpoints included 3P-MACE components, hospitalization for heart failure, revascularizations, and adverse events.

**Results:**

From a background population of 5,242,201 citizens, 330,193 were identified as having diabetes. PSM produced two very well matched cohorts of 4063 patients each, who initiated GLP-1RA or BI after an average of 2.5 other diabetes drug classes. Patients were 63-year-old and only 15% had a baseline history of cardiovascular disease. During a median follow-up of 24 months in the intention-to-treat analysis, 3P-MACE occurred less frequently in the GLP-1RA cohort (HR versus BI 0.59; 95% CI 0.50–0.71; p < 0.001). All secondary cardiovascular endpoints were also significantly in favor of GLP-1RA. Results were confirmed in the as-treated approach and in several stratified analyses. According to the E-value, confounding by unmeasured variables were unlikely to entirely explain between-group differences in cardiovascular outcomes.

**Conclusions:**

Patients with T2D who initiated a GLP-1RA experienced far better cardiovascular outcomes than did matched patients who initiated a BI in the same healthcare system. These finding supports prioritization of GLP-1RA as the first injectable regimen for the management of T2D.

**Supplementary Information:**

The online version contains supplementary material available at 10.1186/s12933-021-01414-3.

## Background

Since its discovery in 1921, insulin has become the mainstay of diabetes management. After 100 years, insulin use is still compulsory for correcting hyperglycemia in individuals with type 1 diabetes. On the other side, pharmaceutical developments have provided several valid alternatives to insulin for the management of type 2 diabetes (T2D). When insulin therapy is required to treat T2D, basal insulin (BI) is the preferred initial approach, because it allows similar glycemic improvement as compared with more intensive regimens, but with lower hypoglycemia risk [1]. For decades, BI has been one of the most common second-line regimen after failure of metformin monotherapy, and the only possible injectable therapy after failure of oral combinations. The latest consensus algorithm for the management of T2D jointly issued by the American Diabetes Association (ADA) and the European Association for the Study of Diabetes (EASD) [2, 3] recommends GLP-1 receptor agonists (GLP-1RA) as the first injectable therapy, before BI, in most patients with T2D. BI remains an established approach for patients with high HbA1c levels (> 97 mmol/mol [> 11%]) and symptoms of hyperglycemia or hypercatabolism.

Randomized controlled trials (RCTs) have shown that, compared to BI, GLP-1RA granted similar or greater HbA1c reductions, but with lower rates of hypoglycemia, substantial weight loss, and easier administration schedule [4]. In addition, cardiovascular outcome trials (CVOTs) have established that, while insulin can be considered safe with regards to major adverse cardiovascular events (MACE) [5, 6], GLP-1RA (liraglutide, semaglutide, dulaglutide, albiglutide, and efpeglenatide) reduce the rates of MACE compared to placebo [7–9]. Furthermore, treatment with GLP-1RA could be more cost-effective than insulin therapy [10]. However, the populations enrolled in trials with BI or GLP-1RA were different, making results not directly comparable. In fact, no trial has compared head-to-head the effects of GLP-1RA versus BI on the rates of MACE.

In the absence of evidence from RCTs, real-world studies on hard outcomes have compared several classes of glucose-lowering medications, including GLP-1RA, dipeptidyl-peptidase 4 inhibitors (DPP-4i), and sodium glucose cotransporter-2 inhibitors (SGLT2i). Patients initiating GLP-1RA under free-living conditions had better cardiovascular outcomes compared to those initiating DPP-4i [11], whereas the comparison between GLP-1RA and SGLT2i yielded mixed results according to the setting and duration of observation [12–15].

Here, we wished to verify if, in routine clinical practice, patients with T2D who initiated a GLP-1RA exhibited better cardiovascular outcomes than similar patients who initiated BI at the same disease stage and with the same degree of comorbidity. To this end, we conducted a Region-wide retrospective study on propensity score matched cohorts of patients.

## Methods

### Study design and data source

This was a Region-wide, retrospective, longitudinal, comparative effectiveness study. The framework for this study has been describe previously [11, 13, 16]. Briefly, data used in this study were extracted from the administrative data repository of the Veneto Region (Italy), which contains all healthcare records of about 5,000,000 citizens on the Region from 2011. Data of biochemical laboratory analyses are available in the regional Health Information Exchange (rHIE) system for a subset of individuals [17]. All data used for this study were anonymized in compliance with Italian law before being used for research purposes. The study protocol conforms to the ethical guidelines of the 1975 Declaration of Helsinki and was approved by the data handling board (Arsenàl.IT). In compliance to national regulations on retrospective studies using routinely accumulated data (Italian Medicines Agency determination 20/03/2008). All patients had provided informed consent to the re-use of medical data for research purposes as a prerequisite for entering the database.

### Cohort identification

The background population of patients with diabetes mellitus was selected among Italian citizens residing in Veneto who had been eligible beneficiaries for at least 1 year between January 1st, 2011 and September 30th, 2018 or time of death. In order to identify individuals with diabetes, we used an algorithm based on claims data and validated against the gold standard clinical diagnosis [18]. From the background population, we selected patients who initiated a GLP-1RA (ATC A10BJ: exenatide, liraglutide, lixisenatide, dulaglutide) or basal insulin (ATC: glargine A10AE04, A10AE54; detemir A10AE05; degludec A10AD06, A10AE06, A10AE56; others A10AC04, A10AD04, A10AD05, A10AC01, A10AD01) within the observation time. New users of such drugs were defined as patients with a new (first) prescription and no prescription of the same drug class (GLP-1RA or BI) in the previous 12 months. The index date was set as the date patients filled their first prescription for GLP-1RA or BI. Use of fast-acting insulin within the preceding 12 months was an exclusion criterion because we wished to compare two different strategies of injectable therapy without other concomitant injectable glucose lowering medication. Initiators switching from another GLP-1RA or another BI were also excluded.

We defined two analytical approaches. In the “ITT” approach, patients were followed from the index date to the event, death, or the last available observation, whichever occurred first. In the “as treated” (AT) approach, the observation was stopped in case patients discontinued treatment with GLP-1RA or BI. Discontinuation was defined as lapse in GLP-1RA or BI prescription longer than 12 months; censoring happened at the end of the 12-month period. The 12-month gap was identified as part of an empirical correlation analysis between discontinuation time as apparent within specialist records (hospital data) vs. administrative claims (the main data used for this study).

### Outcome ascertainment

The primary outcome for this study was a modified version of the 3-point MACE (3P-MACE), defined as occurrence of myocardial infarction, stroke, or all-cause death. Death from any cause was used in this version of the 3P-MACE in place of death from cardiovascular causes because causes of death were not available in the database. Individual components of the 3P-MACE were secondary endpoints, along with additional outcomes: hospitalization for heart failure (HHF), hospitalization for any cardiovascular disease (CVD), and revascularization procedures. To ascertain outcomes, we used ICD-9-CM codes from hospital discharge claims: myocardial infarction (410–414), stroke (431–436), hospitalization for heart failure (428), hospitalization for any cardiovascular cause (390–459), and revascularization (00.55, 00.61–66, 36.03, 36.06–7, 36.10–19, 38.48, 39.50, 39.52, 39.71, 39.90). Time to event was coded in months to comply with the time resolution of anonymized dates of death in the administrative database.

### Adverse events

We evaluated occurrence of a series of adverse events during the observation, which are of particular interest for GLP-1RA and BI: pancreatic cancer (155.1, 156, 157), pancreatitis (577.0), acute and chronic kidney disease (584), diabetic ketoacidosis (250.1), and severe hypoglycemia (250.3, 250.8, 251.0, 251.2). Validity of claims-based diagnosis for pancreatic cancer was confirmed in at least three independent databases [19–21], whereas validation of kidney disease yielded more modest agreement with clinical diagnosis [22, 23].

### Statistical analysis

Data are presented as mean (standard deviation, SD) for continuous variables, or as percentage for categorical variables. Due to the real-world nature of the study, differences in patients’ characteristics between the two groups were expected. To obtain two subgroups of patients that were comparable, we used propensity score (PS) matching (PSM) with the nearest neighbor method and the logit distance, and a maximum caliper 0.5% SD of PS. To estimate the PS, we used a logistic regression model with the following covariates: age at index date, sex, clinical history in the claim database (months between the first available claim and the index date), claims-based diabetes duration (months between the first diabetes-related claim, including the exemption code backfilled before 2011, and the index date, which is a proxy of real diabetes duration); history of a first specialist diabetes visit in the preceding year; concomitant risk factors (hypertension, dyslipidemia), diabetic complications (peripheral artery disease, myocardial infarction, ischemic heart disease, stroke or transient ischemic attack—TIA, heart failure, cardiovascular disease, neurological complications, ocular complications, renal complications, chronic kidney disease—CKD), history of severe hypoglycemia, other comorbidities (chronic pulmonary disease, systemic inflammatory disease, cancer), the Charlson comorbidity (derived from administrative claims as previously described [24, 25]), the number of different A10B-class drugs (“blood glucose lowering drugs, excluding insulins”), use of glucose lowering medications in the year before the index date (metformin, sulfonylureas, SGLT-2i, pioglitazone), and use of other drugs in the year before the index date (ACE inhibitors, diuretics, beta blockers, other blood pressure lowering drugs, statins, fibrates or omega-3, PCSK9 inhibitors, ezetimibe, and anti-platelet agents). Definitions of all these variables using administrative claims are described in Additional file 1: Table S1. Results of laboratory analyses (fasting glucose, HbA1c, total cholesterol, HDL cholesterol, LDL cholesterol, triglycerides, estimated glomerular filtration rate (eGFR) [26]) and body weight were available for a minority of patients in the database. Such data could not be used for PSM, but were checked to verify the overall balance.

After PSM, we used the Mann–Whitney’s U test or the chi-square test or to evaluate the balance in continuous or categorical variables, respectively. The match was considered to be successful when p-values for all comparisons were greater than 0.05 or the differences were small (absolute standardized mean difference < 0.10). This implies that, with large sample size, differences yielding a p-value < 0.05 would be considered non-clinically significant if SMD was < 0.10 (i.e. a difference that is < 10% the pooled SD for continuous variables).

The primary analysis was performed using the ITT approach, comparing hazard ratios (HRs) for GLP-1RA versus BI initiators in terms of 3P-MACE, its individual components, and other secondary outcomes. We performed the following secondary analyses: (i) a repeat of the primary analysis using the AT approach; (ii) a comparison of all endpoints in the ITT dataset stratified by the history of baseline CVD; (iii) a comparison of 3P-MACE in the ITT dataset stratified by a series of baseline characteristics (age, sex, diabetes duration, use of sulfonylureas, statins, renin-angiotensin system blockers, microvascular complications (ocular, neurologic, renal and CKD); (iv) a comparison between exendin or human GLP-1RA versus BI; (v) a re-match of groups excluding patients who initiated exendin-based GLP-1RA. For all analyses, we used Cox regression to estimate HRs and tested statistical significance at the 0.05 level. The E-value was calculated to assess the extent of confounding in attributing causality to the observational association between treatment and outcomes [27, 28].

The statistical package R v4.0.1 and the python packages lifelines v0.21.0 and scipy v1.4.1 were used.

## Results

### Patient cohorts

**Fig. 1:**
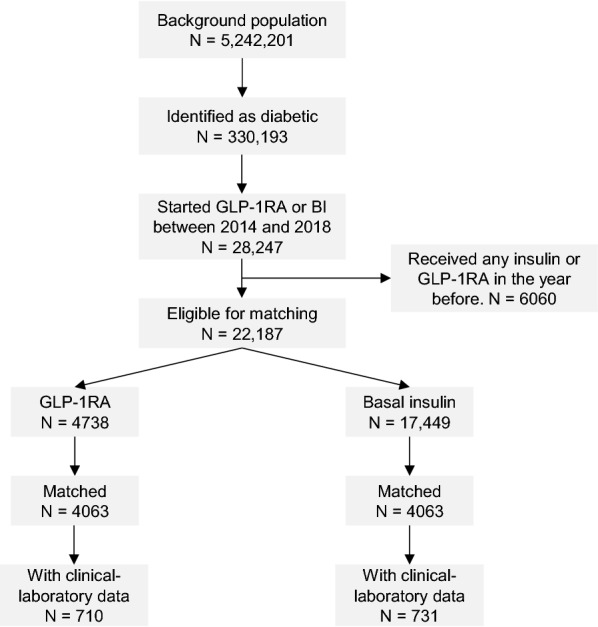
Study flowchart

The initial population of citizens of the Veneto Region was composed of 5,242,201 individuals. According to a validated algorithm, 330,193 could be identified as being affected by diabetes (prevalence 6.3%), 28,247 of whom had started a GLP-1RA or BI between 2014 and 2018. After excluding those treated with any insulin (bolus, intermediate, mixed, or basal) or GLP-1RA in the previous year, we identified 22,187 patients newly treated with GLP-1RA (n = 4738) or BI (n = 17,449). Figure 1 shows the study flow-chart. Patients belonging to these unmatched groups significantly differed for most baseline clinical characteristics. Overall, patients initiating BI were older, had longer diabetes duration and displayed a more advanced disease stage and more comorbidities, including a relatively high prevalence of cardiovascular disease (32%). PSM selected two cohorts of 4063 patients each, who were very well matched (p > 0.05 or SMD < 0.10 for all variables, Table 1 and Additional file 1: Fig. S1). Additional file 1: Fig. S2 shows the distribution of PS before and after matching. Matched patients were on average 63-year-old, 61% males, with an estimated diabetes duration of 9 years. 83% were hypertensive and 71% dyslipidemic. Only 15% were affected by cardiovascular disease. The vast majority of patients were on metformin (90%), whereas a sulphonylurea or a DPP-4i were used in 53% and 41% of patients, respectively. As a result, patients were initiating GLP-1RA or BI on top of an average of 2.5 classes of glucose lowering medications. A large proportion of patients were taking RAS blockers (71%) and statins (62%). GLP-1RA were distributed as follows: dulaglutide 43%, liraglutide 35%, exenatide 18%, lixisenatide 4%. BI were distributed as follows: glargine 79%, detemir 10%, degludec 4%, others 7%. As determined in a small subset of patients (17% in the GLP-1RA group and 18% in the BI group), average baseline HbA1c was 7.7% in the GLP-1RA group and 8.3% in the BI group, whereas body weight was 94.9 kg in the GLP-1RA group and 84.7 kg in the BI group. Blood pressure, lipids, and eGFR were similar between the two subgroups (Additional file 1: Table S2).

**Table 1:** Baseline clinical characteristics before and after matching

	Before matching				After matching			
	GLP-1RA (N = 4738)	Insulin (N = 17,449)	SMD*	p value**	GLP-1RA (N = 4,063)	Insulin (N = 4,063)	SMD*	p value**
Demographics								
Age at index date (years)	62.2 (9.4)	71.9 (11.5)	− 0.88	< 0.001	63.3 (9.1)	63.1 (11.2)	0.02	0.124
Female sex (%)	39.3	42.9	− 0.07	< 0.001	39.5	39.1	0.01	0.733
Claims-based history length^a^ (months)	54.4 (18.6)	46.6 (19.3)	0.41	< 0.001	53.0 (18.9)	52.8 (18.5)	0.01	0.152
Claims-based diabetes duration^b^ (months)	105.3 (59.4)	123.5 (66.3)	− 0.28	< 0.001	107.5 (60.5)	108.5 (60.2)	− 0.02	0.201
Recent outpatient prescription for first endocrinological visit	17.8	24.8	− 0.17	< 0.001	19.1	19.6	− 0.01	0.594
Risk factors								
Hypertension (%)	83.7	88.4	− 0.14	< 0.001	83.7	82.5	0.03	0.164
Dyslipidaemia (%)	71.9	68.0	0.08	< 0.001	71.3	70.9	0.01	0.750
Macrovascular complications								
Peripheral circulatory complications (%)	1.0	5.0	− 0.20	< 0.001	1.2	1.5	− 0.03	0.282
Infarction (%)	5.4	13.7	− 0.26	< 0.001	5.8	6.0	− 0.01	0.778
Ischemic heart disease (%)	9.8	19.2	− 0.25	< 0.001	10.5	10.4	0.00	0.913
Stroke or TIA (%)	3.1	8.6	− 0.21	< 0.001	3.3	4.1	− 0.04	0.078
Heart failure (%)	1.9	12.4	− 0.35	< 0.001	2.1	2.9	− 0.05	0.019
Cardiovascular disease (%)	13.6	32.3	− 0.42	< 0.001	14.5	15.6	− 0.03	0.215
Microvascular complications								
Neurological complications (%)	0.1	1.1	− 0.11	< 0.001	0.1	0.3	− 0.04	0.099
Ocular complications (%)	0.2	0.6	− 0.06	< 0.001	0.2	0.3	− 0.02	0.382
Renal complications (%)	0.2	2.0	− 0.15	< 0.001	0.2	0.2	0.01	1.000
Chronic kidney disease (%)	1.4	8.5	− 0.28	< 0.001	1.5	1.7	− 0.02	0.430
Severe hypoglycaemia (%)	0.3	2.3	− 0.15	< 0.001	0.3	0.6	− 0.04	0.108
Comorbidities								
Chronic pulmonary disease (%)	32.1	32.1	0.00	0.981	31.7	30.2	0.03	0.143
Systemic inflammatory disease (%)	2.3	1.9	0.03	0.083	2.3	2.3	0.00	0.882
Cancer (%)	10.1	17.6	− 0.20	< 0.001	10.7	11.2	− 0.02	0.456
Charlson comorbidity index	0.3 (0.9)	1.3 (1.9)	− 0.52	< 0.001	0.3 (1.0)	0.4 (1.0)	− 0.08	< 0.001
0, n (%)	3902 (82.4)	9479 (54.3)			3318 (81.7)	3171 (78.0)		
1, n (%)	443 (9.3)	2466 (14.1)			391 (9.6)	440 (10.8)		
2, n (%)	244 (5.1)	2276 (13.0)			221 (5.4)	284 (7.0)		
3+, n (%)	149 (3.1)	3228 (18.5)			133 (3.3)	168 (4.1)		
Glucose lowering medications								
No. of different A10B therapies^c^	2.5 (1.2)	2.4 (1.2)	0.08	< 0.001	2.5 (1.3)	2.5 (1.3)	− 0.02	0.290
Metformin (%)	90.6	79.0	0.30	< 0.001	89.6	89.9	− 0.01	0.742
Sulfonylureas (%)	48.6	72.8	− 0.53	< 0.001	53.1	53.8	− 0.02	0.505
SGLT2i (%)	5.5	1.8	0.23	< 0.001	4.8	4.7	0.00	0.917
DPP4i (%)	43.0	27.7	0.33	< 0.001	40.7	40.6	0.00	0.982
Pioglitazone (%)	16.4	9.1	0.24	< 0.001	15.0	15.2	− 0.01	0.828
Other therapies								
ACE inhibitors (%)	71.9	71.8	0.00	0.912	71.4	70.8	0.01	0.590
Diuretics (%)	17.5	40.6	− 0.49	< 0.001	18.9	18.1	0.02	0.361
Beta blockers (%)	33.9	43.7	− 0.20	< 0.001	35.1	34.2	0.02	0.401
Other antihypertensives (%)	8.2	10.4	− 0.07	< 0.001	8.4	7.9	0.02	0.465
Statins (%)	62.8	59.5	0.07	< 0.001	62.1	61.5	0.01	0.553
Fibrates or omega-3 (%)	11.8	9.9	0.06	< 0.001	11.6	11.7	− 0.00	1.000
PCSK9 inhibitors	0.0	0.0	0	1.000	0.0	0.0	0	1.000
Ezetimibe (%)	3.3	1.6	0.13	< 0.001	2.7	2.6	0.01	0.729
Platelet aggregation inhibitors (%)	33.2	47.4	− 0.29	< 0.001	34.5	34.2	0.01	0.797

Therapy variables were calculated starting from 12 months before the index date, unless otherwise indicated. Pre-existing conditions were calculated with all available data up to the index date. Clinical-laboratory data refer to the visit closest to the index date. Absolute SMD values are shown

*Standardized mean differences (positive if SGLT2i greater; “< 0.01” if |SMD| < 0.01)

**Chi-squared test for dichotomous variables (expressed as %), Mann–Whitney’s U test otherwise

^a^Time interval between the first available claim and the index date

^b^Time interval between the first claim or exemption from co-payment indicating diabetes and the index date

^c^Computed using all available data up to the index date

### Outcome analysis

**Fig. 2:**
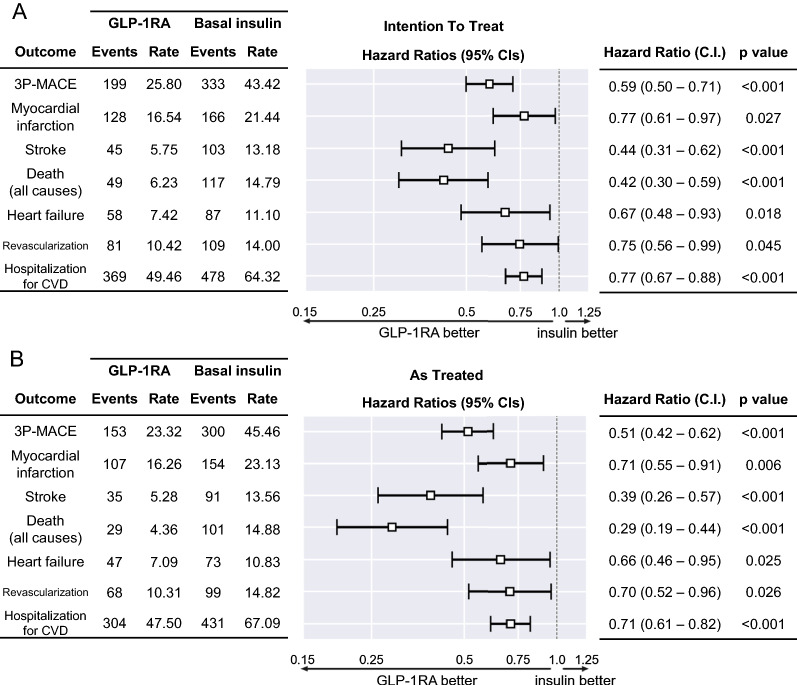
Comparative cardiovascular outcomes. The Forest plot shows hazard ratios (HR) and 95% confidence intervals (CI) of the primary endpoint 3P-MACE (3-point major adverse cardiovascular events), its components and other cardiovascular disease (CVD) outcomes according to the primary intention-to-treat analysis (**A**) or the as-treated (**B**) analysis. HR < 1.0 are indicative of lower event rates in the GLP-1RA group than in the BI group. Number of events and event rates (/1000 patient year) are also reported

**Fig. 3:**
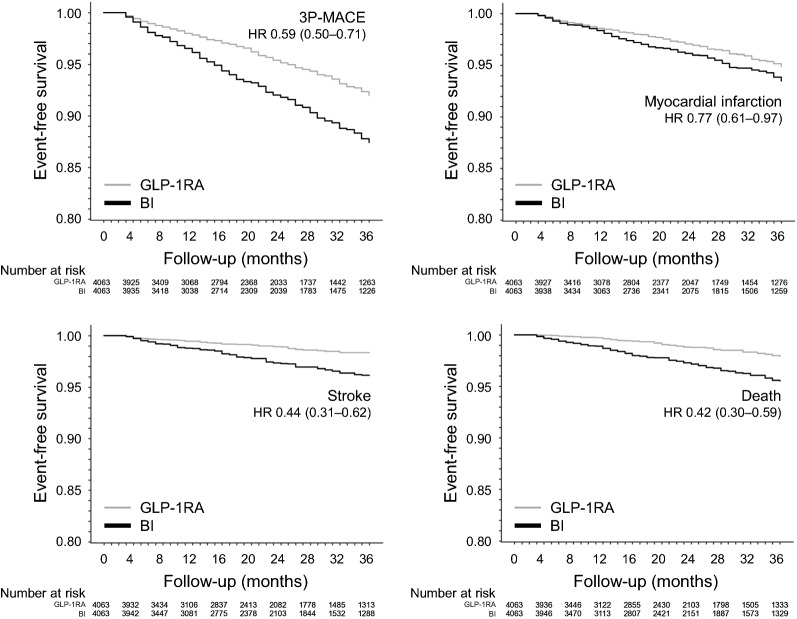
Kaplan–Meier curves. Even-free survival curves in the two marched groups of GLP-1RA and BI initiators are shown for the primary composite outcome and its components. The hazard ratio (HR) and 95% CI are reported. Number of patients at risk are also shown for each timepoint

The median duration of follow-up was 24 (IQR: 12–36) months with the ITT approach, 17 months (IQR: 12–30) with the AT approach. In the primary analysis (ITT), we recorded 532 3P-MACE, 199 among patients who initiated GLP-1RA (25.8 events/1000 patient year) and 333 among patients who initiated BI (43.4 events/1000 patient year). The corresponding HR was 0.59 (95% CI 0.50–0.71; p < 0.001) in favor of the GLP-1RA group (Fig. 2A). Among secondary outcomes, patients who newly initiated GLP-1RA, as compared to those who newly initiated BI, had lower rates of all-cause death (HR 0.42; 95% CI 0.30–0.59; p < 0.001), myocardial infarction (HR 0.77; 95% CI 0.61–0.97; p = 0.027), stroke or TIA (HR 0.44, 95% CI 0.31–0.62; p < 0.001), HHF (HR 0.67; 95% CI 0.48–0.93; p = 0.018), revascularization (HR 0.75, 95% CI 0.56–0.99; p = 0.045), and hospitalization for any cardiovascular cause (HR 0.77; 95% CI 0.67–0.88; p < 0.0001). Results of the ITT analysis were confirmed using the AT approach, with HR point estimates moving to the left, more in favor of GLP-1RA versus BI group for all endpoints (Fig. 2B). Kaplan–Meier curves for 3P-MACE and its components are shown in Fig. 3.

### Subgroup analyses

**Fig. 4.**
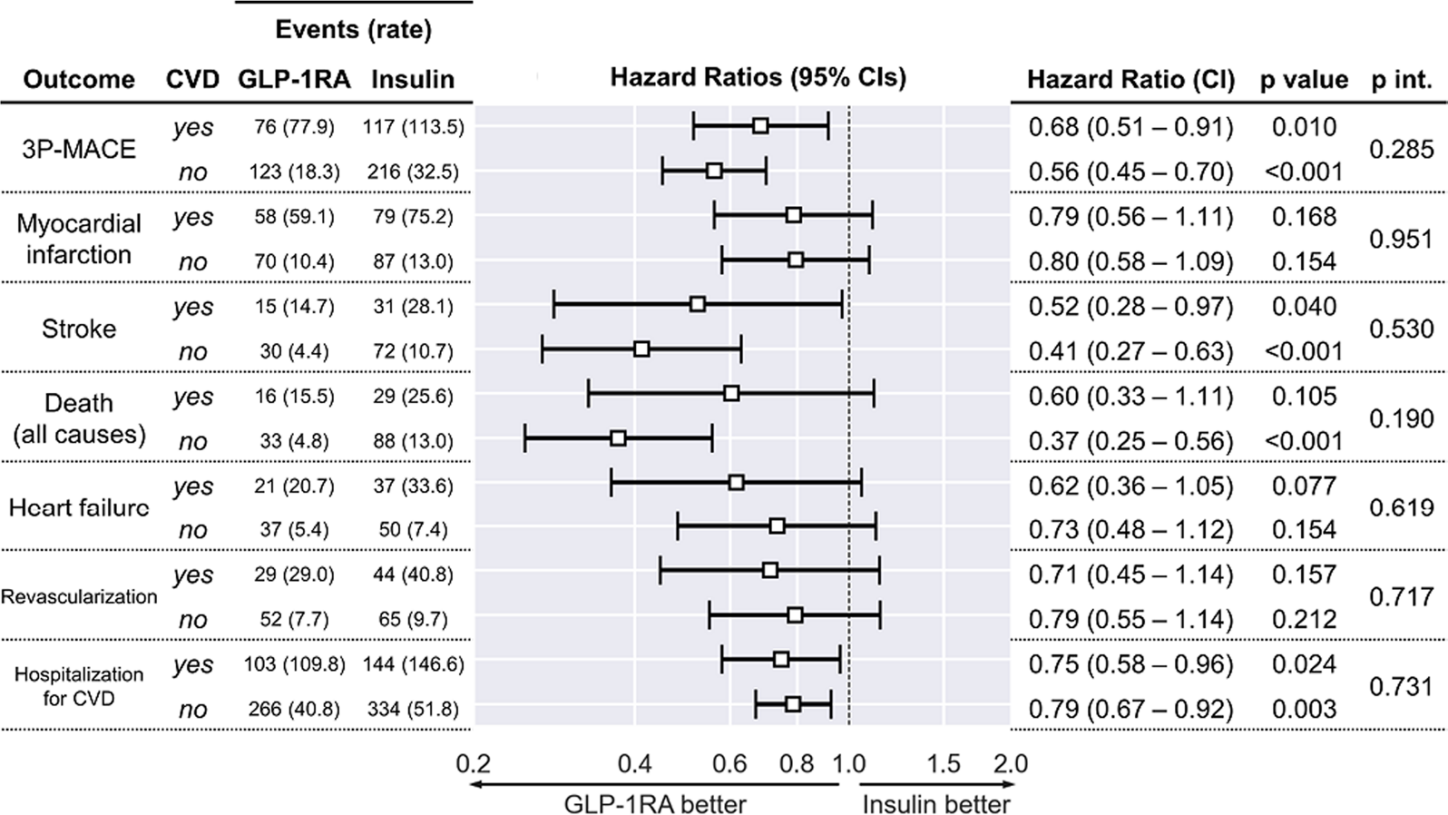
3P-MACE by baseline CVD history. The forest plot shows hazard ratios (HR) and 95% confidence intervals (CI) of all study endpoints in patients stratified within each group by presence or absence of CVD at baseline. The numbers of events in each subgroup and the corresponding rate (between brackets, in events/1000 person-years) are reported. In addition to p values of the HR in each subgroup, the interaction term (group × CVD) p value (p int.) is also reported. Subject distribution was as follows: 591 GLP-1RA initiators with CVD vs. 3472 without; 632 basal insulin initiators with CVD vs. 3431 without

**Fig. 5.**
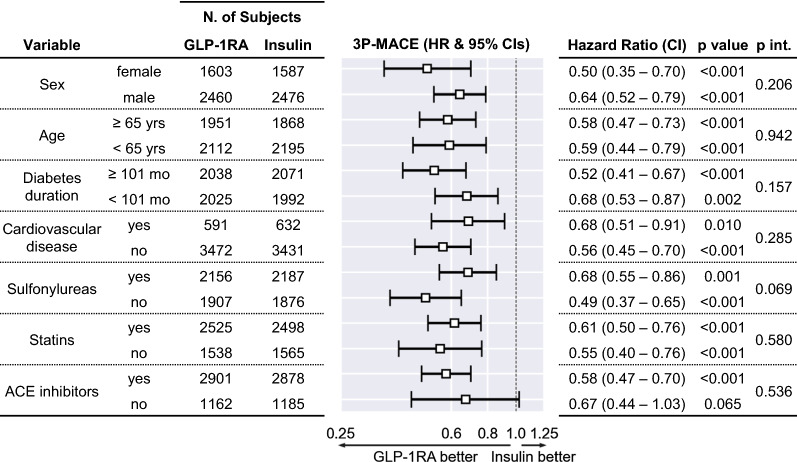
3P-MACE by baseline covariates. The Forest plot shows hazard ratios (HR) and 95% confidence intervals (CI) of the primary endpoint in patients stratified within each group by key baseline characteristics. The numbers of patients in each subgroup are reported. In addition to p values of the HR in each subgroup, the interaction term (group × covariate) p value (p int.) is also reported

The rates of 3P-MACE were significantly lower among patients who initiated GLP-1RA versus BI independently of baseline CVD (Fig. 4). This was also the case for the rates of stroke and hospitalization for cardiovascular causes. The HR for all-cause death was significantly in favor of GLP-1RA only in the absence of baseline CVD, but we detected no significant interaction between a baseline history of CVD and the HR for any of the endpoints. Further stratification of the analysis of the primary outcome by other baseline characteristics yielded consistent results (Fig. 5): the HR for 3P-MACE was always in favor of GLP-1RA versus BI independently of age category, sex, duration of diabetes, and concomitant medications, except in people who were not taking ACE inhibitors, for whom the confidence interval crossed unity. The number of subjects and, consequently, the rate of events in people with pre-existing microvascular disease was too low for the univariate Cox model to converge. Outcomes were in favor of GLP-1RA versus BI independently of the former’s human or exendin origin (Additional file 1: Fig. S3). After rerunning the analysis and excluding exendin-based GLP-1RA initiators before PSM, results were consistent with the rest of our findings: the HR associated with 3P-MACE was 0.62 (95% CI 0.50, 0.76; p < 0.0001) in favor of GLP-1RA.

The E-value for 3P-MACE was 2.78 for the point-estimate and 2.17 for the upper CI bound. This means that an unmeasured confounder would need to have a hazard ratio ≥ 2.17 with both the treatment and the outcome to make the observed treatment-outcome association no longer significant, after adjusting for measured covariates [28]. The corresponding values for all-cause death would be 4.19 and 2.78.

### Adverse events

We examined occurrence of a series of adverse events of interest (Additional file 1: Table S3). There were 39 cases of pancreatic cancer, 11 in the GLP-1RA group and 28 in the BI group, equal to a HR of 0.39 (95% CI 0.20–0.79; p = 0.009). Cases of pancreatitis were nominally lower during new therapy with GLP-1RA (n = 3) than with BI (n = 9) but not significantly (p = 0.101). The number of renal failure events (acute and chronic) were nominally lower in the GLP-1RA group than in the BI group (p = 0.379). Severe hypoglycemia occurred less frequently after initiation of GLP-1RA than after initiation of BI (32 versus 50 events, HR 0.56, p = 0.052). No difference in the rates of fractures was detected.

## Discussion

In this Region-wide retrospective study, we found that patients with T2D who initiated a GLP-1RA under routine care between 2011 and 2018 had better cardiovascular outcomes than matched patients who initiated a BI in the same period and healthcare system.

Although there is no direct comparative trial on the cardiovascular outcomes of GLP-1RA and BI, our findings are consistent with results of CVOTs testing GLP-1RA or BI against placebo. The ORIGIN trial demonstrated cardiovascular safety of insulin glargine versus placebo in patients with cardiovascular risk factors and pre-diabetes or T2D [5]. Subsequently, the DEVOTE trial demonstrated that, among insulin-naïve T2D patients at high risk for cardiovascular events, degludec was noninferior to glargine with respect to the incidence of MACE, but was associated with significantly lower rates of severe hypoglycemia [6]. Of note, severe hypoglycemia was associated with adverse outcomes, projecting the risk over at least 1 year [29].

On the other side, placebo-controlled trials on GLP-1RA have shown that these drugs, most likely as a class, reduced the rates of MACE, including mortality and events attributable to atherothrombotic disease, such as MI and stroke [9]. In parallel to this evidence from trials, it is re-assuring that, even under routine care, our results show that initiation of GLP-1RA is associated with better cardiovascular outcomes than initiation of BI. Therefore, our data support prioritization of GLP-1RA as the first injectable regimen for the management of T2D that is envisaged by modern treatment algorithms [2, 3]. It is remarkable that the benefit of GLP-1RA versus BI was evident in patients with T2D and a smaller prevalence of baseline cardiovascular disease (15%) than in trials (30–100%). Results were highly consistent in subgroup analysis, confirming better cardiovascular outcomes of patients in the GLP-1RA group independently from age, sex, a history of cardiovascular disease at baseline, and other stratification variables. Despite our recent finding that patients initiated on human-based GLP-1RA had better outcomes than those initiated on exendin-based GLP-1RA [16], initiation of either type of GLP-1RA was associated with better outcomes than initiation of BI and a sensitivity analysis limited to human-based GLP-1RA yielded similar results as in the primary analysis. It should be noted that the recent AMPLITUDE-O trial demonstrated superiority of the exendin-based GLP-1RA efpeglenatide over placebo on cardiovascular and renal outcomes among people with type 2 diabetes and cardiovascular or renal disease or multiple risk factors [7]. Therefore, the hypothesis that the molecular origin of GLP-1RA determines its cardiovascular protective effects hardly holds up after AMPLITUDE-O.

The relative reduction in hazard we observed among patients who initiated a GLP-1RA versus a BI was particularly strong for stroke and mortality (exceeding 50%), but was statistically significant and clinically relevant also for MI and HHF. Of note, when we limited the observation to the period effectively covered by drug prescription (AT approach), outcomes were even more in favor of GLP-1RA versus BI, with a 71% lower relative hazard of death. This result supports the biological plausibility of our findings, because limiting the observation to the period when patients were actually taking GLP-1RA or BI should indeed uncover the true difference between the two approaches.

In the literature, we found a striking scarcity of studies reporting a similar observational comparison between GLP-1RA and BI. In the analysis of a large U.S. insurance claims database (2011–2015), use of GLP-1RA was associated with lower and use of BI was associated with higher rates of cardiovascular events when compared to use of DPP-4i, but no direct comparison was performed between injectable strategies [30]. In a smaller retrospective study from Taiwan (2012–2016), patients who received liraglutide had a 35% relative lower risk of a composite cardiovascular outcome than those receiving BI [31], which was particularly strong for all-cause mortality and stroke, as in our study.

We acknowledge that typical biases occur when retrospectively comparing the outcomes associated with use of insulin versus those associated with use of non-insulin therapies. This is because patients with T2D who receive insulin are supposedly frailer than those using non-insulin drugs [32]. Usually, insulin is prescribed to patients with a longer diabetes duration, worse glycemic control, more advanced disease stage and beta-cell dysfunction with failure of prior diabetes drugs, more cardiac, renal, and hepatic comorbidities, advanced cancer, infections, or acute conditions. In order to limit the impact of such confounders, in our study, we matched patients for a series of important variables that can drive differential outcomes, including the estimated diabetes duration, the number of diabetes drug classes, all major comorbidities, and concomitant medications. In addition, we excluded all patients who, prior to the index date, had received any prescription for bolus insulin, which is a proxy of patient’s frailty more than BI. PSM allowed a comparison of similar patients who initiated GLP-1RA or BI, but it should be kept in mind that BI initiators under routine care were sicker. Therefore, our results should not be generalized to all patients who initiate BI in clinical practice. A major limitation is that our study was based on an administrative claim database, which typically lacks clinical-level patients’ data and allow a partial representation of the patients’ health status. Indeed, clinical-laboratory data were available for a small proportion of patients (< 20%), implying we could not match for HbA1c, BMI, blood pressure and other clinical variables that are usually associated with cardiovascular outcomes. In < 20% of participants from the matched cohorts, we found reasonable match in risk factors such as blood pressure, lipid profile and eGFR. If PSM had been unsuccessful in translating administrative claims balance into clinical data balance, we would have seen a strong imbalance in clinical data. On the contrary, we found that the subsampled cohorts showed matching in some clinical parameters, and, while this is no definitive proof of perfect balance in clinical data, it demonstrates that our PSM procedure was good enough to induce at least that amount of matching. On the other side, HbA1c was 0.6% higher in those initiating BI than in those initiating GLP-1RA. This difference is due at least in part to the existence of an upper HbA1c limit for reimbursement of GLP-1RA initiation therapy in Italy during the study period. Because of such restriction, patients with HbA1c > 9% were excluded from GLP-1RA reimbursement and were therefore more likely to initiate BI. In the absence of a formal match in such clinical-level data on risk factors, is possible that the higher HbA1c drove at least part of the worse cardiovascular outcomes of patients in the BI group [33, 34]. At the same time, patients initiating a GLP-1RA had a 10 kg higher average body weight. Since adiposity is a major driver of cardiovascular events in T2D [35], it is notable that the rates of cardiovascular events were lower on GLP-1RA than on BI, despite the markedly higher body weight. In this regards, weight loss that usually follows initiation of GLP-1RA could contribute to the better cardiovascular outcomes, while initiation of BI is usually accompanied by weight gain [36]. Furthermore, the trend lower rates of severe hypoglycemia in the GLP-1RA group could have contributed to the better cardiovascular outcomes in those patients, because severe hypoglycemia is strongly associated with, and can precipitate, cardiovascular events [37]. Concerning the analysis of adverse events, we acknowledge that some risk factors for pancreatic disease (e.g., obesity, alcohol use and gallbladder disease) could not be matched for. In addition, the number of events was small leading to large confidence intervals of the estimate. Therefore, caution should be paid when interpreting these findings.

As noted above, the lack of key clinical variables remains the major limitation of our study, which amplifies the ever-holding confounding by indication in observational research. Yet, the HR for all-cause mortality associated with GLP-1RA versus BI was close to the point where confounding is generally considered unlikely to be a major issue [32]. Consistently, calculation of E-values for the primary endpoint and mortality in the ITT analysis yielded particularly robust estimates. Based on results of CVD prediction models developed in T2D, it seems unlikely that our analysis is missing one or more unmeasured confounders associated with both treatment and the outcome with a risk ratio > 2. For example, higher HbA1c is usually associated with worse CVD outcomes and HbA1c was significantly 0.6% higher among BI versus GLP-1RA initiators. However, in two models for MACE prediction among people with T2D, the HR associated with 1% increase in HbA1c was 1.11 and 1.12 [38, 39]. Therefore, differences in baseline glucose control are unlikely to explain the higher 3P-MACE rate after BI initiation compared to GLP-1RA initiation, and even more so for all-cause mortality. Additionally, the higher body weight among GLP-1RA initiators would counterbalance the lower HbA1c, with a similar HR for cardiovascular events [38].

Our study has other notable strengths as it represents, so far, the largest and most contemporary comparison of cardiovascular outcomes of patients treated with GLP-1RA versus BI under routine care, using state-of-the-art methods for comparative observational research.

## Conclusion

Patients with T2D who initiated a GLP-1RA experienced far better cardiovascular outcomes than did matched patients who initiated a BI in the same Region and healthcare system. Although part of this striking difference could be due to residual confounding by indication, our findings support the consensus on the use of GLP-1RA as the first injectable therapy for the management of T2D. In parallel, we acknowledge that the GLP-1RA/BI association is a valuable option in the management of T2D, either as fixed-ratio or loose combination. It can be helpful in the subsequent steps of therapeutic intensification and to simplify regimens as an alternative to basal-bolus insulin [40, 41]. The widespread use of weekly injectable GLP-1RA has improved acceptability of this therapy. With the availability of oral GLP-1RA, overcoming the injection barrier has the potential to unlock the benefits of GLP-1RA for a larger number of people with T2D. However, the cardiovascular benefits of oral semaglutide may not fully be appreciated until the completion of the SOUL trial [42].

## Supplementary Information

**Additional file 1: Table S1.** Claims-based definition of study variables. Each variable was defined as the presence of at least one of the claims-based indicators in the corresponding row. Medication names have been internally mapped 1:1 to ATC codes, exemptions from copayment to regional exemption codes. **Table S2.** Available clinical-laboratory variables for the subset of patients in the matched cohorts. Standardized mean differences (SMD) are shown along with p-values. **Table S3.** Adverse events. Results of Cox regression on adverse events, and corresponding event rates (calculated/1000 person-years). HR are calculated for SGLT2i vs. DPP4i initiators. GUTI, genitourinary tract infections. **Figure S1.** Between group balance before and after matching. The plot shows the absolute standardized mean difference between the group of patients who initiated GLP-1RA or BI for each variable before and after propensity score matching. Matching achieved a robust balance between groups, as evident from a differences < 10% for all variables. **Figure S2.** Distribution of propensity scores. Distribution of propensity scores (PS) is shown for initiators of GLP-1RA and basal insulin in the cohorts before and after propensity score matching (PSM). Note that the sum of the area under curves is always equal to 1.0, despite different sample size before and after PSM. **Figure S3.** Comparative cardiovascular outcomes by type of GLP-1RA. The Forest plot shows hazard ratios (HR) and 95% confidence intervals (CI) of all study endpoints in patients who initiated BI versus those who initiated a human- or exendin-based GLP-1RA. The numbers of patients in each subgroup are reported. In addition to p values of the HR in each subgroup, the interaction term (group × CVD) p value (p int.) is also reported.

## Data Availability

The datasets used and analyzed during the current study are available from the corresponding author on reasonable request.

## Abbreviations

ACE: Angiotensin converting enzyme
ADA: American Diabetes Association
AT: As treated
ATC: Anatomic therapeutic classification
BI: Basal insulin
CI: Confidence interval
CKD: Chronic kidney disease
CVD: Cardiovascular disease
DPP-4i: Dipeptidyl peptidase-4 inhibitors
EASD: European Association for the Study of Diabetes
eGFR: Estimated glomerular filtration rate
GLP-1RA: Glucagon-like peptide 1 receptor agonist
HDL: High density lipoprotein
HHF: Hospitalization for heart failure
HR: Hazard ratio
ICD: International classification of disease
IQR: Interquartile range
ITT: Intention to treat
LDL: Low density lipoprotein
MACE: Major adverse cardiovascular events
PS: Propensity score
PSM: Propensity score matching
rHIE: Regional Health Information Exchange
SD: Standard deviation
SGLT2i: Sodium glucose co-transporter 2 inhibitors
SMD: Standardized mean difference
T2D: Type 2 diabetes
TIA: Transient ischemic attack
