# Supplementary material for: Cardiovascular outcomes after initiating GLP-1 receptor agonist or basal insulin for the routine treatment of type 2 diabetes: a region-wide retrospective study
Source: Cardiovasc Diabetol. 2021 Nov 13;20:222. doi: 10.1186/s12933-021-01414-3 (PMC8590792; doi:10.1186/s12933-021-01414-3)
Supplement: Supplementary file 1 — Additional file 1: Table S1. Claims-based definition of study variables. Each variable was defined as the presence of at least one of the claims-based indicators in the corresponding row. Medication names have been internally mapped 1:1 to ATC codes, exemptions from copayment to regional exemption codes. Table S2. Available clinical-laboratory variables for the subset of patients in the matched cohorts. Standardized mean differences (SMD) are shown along with p-values. Table S3. Adverse events. Results of Cox regression on adverse events, and corresponding event rates (calculated/1000 person-years). HR are calculated for SGLT2i vs. DPP4i initiators. GUTI, genitourinary tract infections. Figure S1. Between group balance before and after matching. The plot shows the absolute standardized mean difference between the group of patients who initiated GLP-1RA or BI for each variable before and after propensity score matching. Matching achieved a robust balance between groups, as evident from a differences < 10% for all variables. Figure S2. Distribution of propensity scores. Distribution of propensity scores (PS) is shown for initiators of GLP-1RA and basal insulin in the cohorts before and after propensity score matching (PSM). Note that the sum of the area under curves is always equal to 1.0, despite different sample size before and after PSM. Figure S3. Comparative cardiovascular outcomes by type of GLP-1RA. The Forest plot shows hazard ratios (HR) and 95% confidence intervals (CI) of all study endpoints in patients who initiated BI versus those who initiated a human- or exendin-based GLP-1RA. The numbers of patients in each subgroup are reported. In addition to p values of the HR in each subgroup, the interaction term (group × CVD) p value (p int.) is also reported. [file 12933_2021_1414_MOESM1_ESM.docx]

*Longato et al.*

Cardiovascular outcomes after initiating a GLP-1 receptor agonist or basal insulin for the routine treatment of type 2 diabetes. A region-wide retrospective real-world study

**Online Appendix**

**Table S1**. Claims-based definition of study variables. Each variable was defined as the presence of at least one of the claims-based indicators in the corresponding row. Medication names have been internally mapped 1:1 to ATC codes, exemptions from copayment to regional exemption codes.

| **Variable** | **ICD-9-CM Diagnosis Codes** | **ICD-9-CM Procedure Codes** | **Medications** | **Exemptions from Copayment** |
| --- | --- | --- | --- | --- |
| **Hypertension** | 401-405 |  | ACE inhibitors, diuretics, beta blockers, other antihypertensives | Hypertension |
| **Dyslipidaemia** |  |  | Statins, fibrates, omega 3, PCSK9 inhibitors, ezetimibe | Dyslipidaemia |
| **Peripheral circulatory complications** | 440, 250.7 | 00.55, 38.48, 39.50, 39.52, 39.71, 39.90 |  |  |
| **Myocardial Infarction** | 410-414 |  |  |  |
| **Ischemic heart disease** | 410-414 | 00.66, 36.03, 36.06, 36.07, 36.10, 36.19 |  | Ischemic heart disease |
| **Stroke or TIA** | 431-436 | 00.61-00.65 |  | Stroke or TIA |
| **Heart failure** | 428 |  |  | Heart failure |
| **Cardiovascular disease** | 410-414, 428, 431-436, 440 | 00.55, 00.61-00.66, 36.03, 36.06, 36.07, 36.10, 36.19, 38.48, 39.50, 39.52, 39.71, 39.90 |  | Ischemic heart disease, stroke or TIA, heart failure |
| **Neurological complications** | 250.6 |  |  |  |
| **Ocular complications** | 250.5 |  |  |  |
| **Renal complications** | 250.4 |  |  |  |
| **Chronic kidney disease** | 585-587 |  |  | Chronic kidney disease |
| **Severe hypoglycaemia** | 250.3, 250.8, 250.10, 250.12 |  |  |  |
| **Chronic pulmonary disease** | 491-496, 518.83, 518.84 |  | Medications for obstructive airway diseases | Chronic pulmonary disease, asthma |
| **Systemic inflammatory disease** |  |  |  | Rheumatoid arthritis, systemic lupus erythematosus, connective tissue disease, Sjogren's syndrome, psoriasis, ankylosing spondylitis |
| **Cancer** |  |  |  | Cancer |

**Table S2**. Available clinical-laboratory variables for the subset of patients in the matched cohorts. Standardized mean differences (SMD) are shown along with p-values.

| **Variable** | **GLP-1RA** | **BI** | **SMD** | **p value** |
| --- | --- | --- | --- | --- |
| Number | 710 | 731 | - | - |
| Body weight, kg | 94.9 (18.9) | 84.7 (16.8) | 0.6 | <0.001 |
| HbA1c, % | 7.7 (0.8) | 8.3 (1.1) | -0.6 | <0.001 |
| Systolic blood pressure, mm Hg | 140.8 (18.8) | 140.3 (19.3) | 0.0 | 0.339 |
| Diastolic blood pressure, mm Hg | 81.3 (11.2) | 80.5 (10.4) | 0.1 | 0.05 |
| Total cholesterol, mg/dl | 169.2 (36.7) | 170.6 (38.3) | 0.0 | 0.345 |
| HDL cholesterol, mg/dl | 48.0 (12.7) | 48.1 (13.7) | 0.0 | 0.437 |
| LDL-cholesterol, mg/dl | 93.6 (32.1) | 95.3 (34.6) | -0.1 | 0.268 |
| Triglycerides, mg/dl | 139.6 (54.5) | 138.5 (59.6) | 0.0 | 0.177 |
| eGFR, ml/min/1.73 m^2^ | 82.7 (18.0) | 82.4 (22.5) | 0.0 | 0.256 |

**Table S3**. Adverse events. Results of Cox regression on adverse events, and corresponding event rates (calculated /1000 person-years). HR are calculated for SGLT2i vs. DPP4i initiators. GUTI, genitourinary tract infections.

|  | **GLP-1RA** | | **Basal insulin** | | **Total** | | **HR (CI)** | **p value** |
| --- | --- | --- | --- | --- | --- | --- | --- | --- |
|  | **Events** | **Rate** | **Events** | **Rate** | **Events** | **Rate** |  |  |
| **Severe hypoglycemia** | 18 | 2.29 | 32 | 4.06 | 50 | 3.18 | 0.56 (0.32-1.00) | 0.052 |
| **Pancreatic cancer** | 11 | 1.40 | 28 | 3.54 | 39 | 2.47 | 0.39 (0.20–0.79) | 0.009 |
| **Pancreatitis** | 3 | 0.38 | 9 | 1.14 | 12 | 0.76 | 0.34 (0.09–1.24) | 0.101 |
| **Acute renal disease** | 35 | 4.46 | 43 | 5.46 | 78 | 4.96 | 0.82 (0.52–1.28) | 0.379 |
| **Fracture** | 47 | 6.01 | 58 | 7.39 | 105 | 6.70 | 0.82 (0.56–1.20) | 0.299 |

**Figure S1.** Between group balance before and after matching. The plot shows the absolute standardized mean difference between the group of patients who initiated GLP-1RA or BI for each variable before and after propensity score matching. Matching achieved a robust balance between groups, as evident from a differences <10% for all variables.

**Figure S2.** Distribution of propensity scores. Distribution of propensity scores (PS) is shown for initiators of GLP-1RA and basal insulin in the cohorts before and after propensity score matching (PSM). Note that the sum of the area under curves is always equal to 1.0, despite different sample size before and after PSM.

**
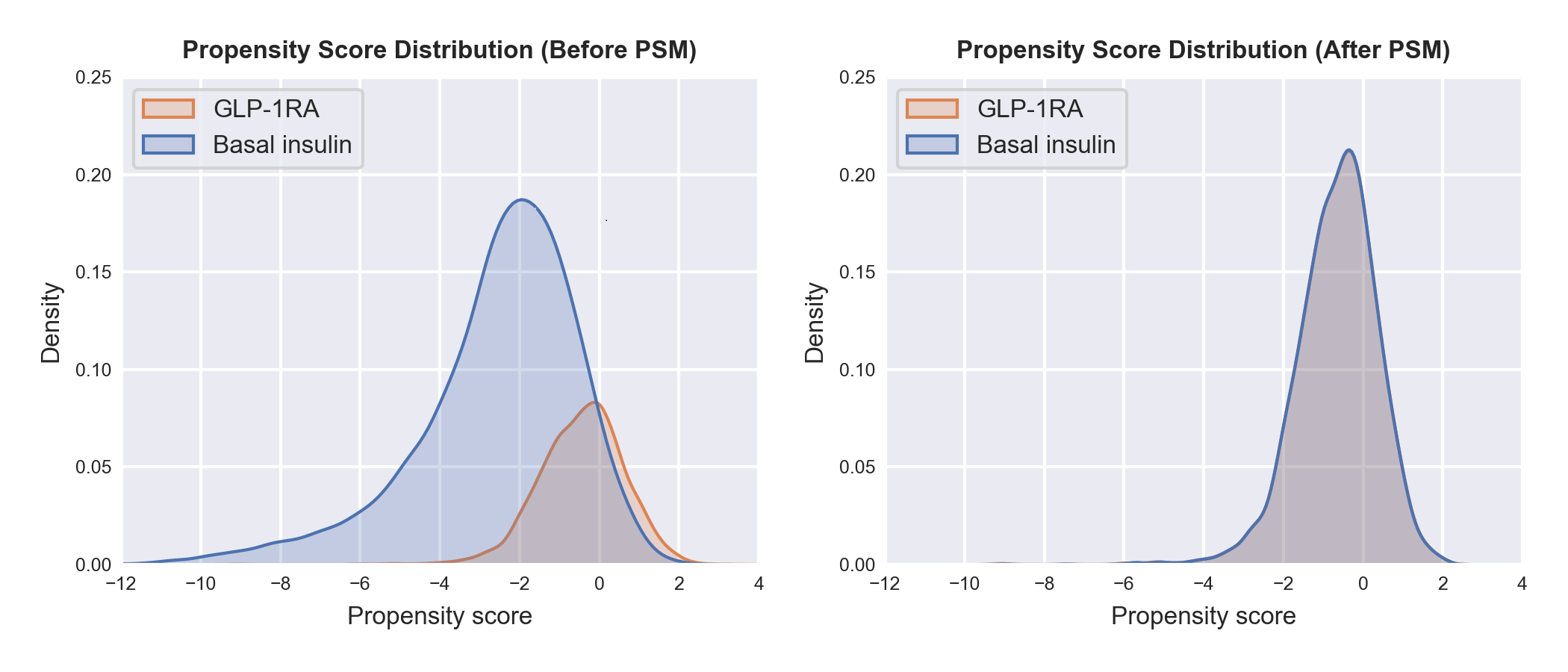
**

**Figure S3**. Comparative cardiovascular outcomes by type of GLP-1RA. The Forest plot shows hazard ratios (HR) and 95% confidence intervals (C.I.) of all study endpoints in patients who initiated BI versus those who initiated a human- or exendin-based GLP-1RA. The numbers of patients in each subgroup are reported. In addition to p values of the HR in each subgroup, the interaction term (group x CVD) p value (p int.) is also reported.
